# Supplementary material for: Psychological Wellbeing of Parents with Infants Admitted to the Neonatal Intensive Care Unit during SARS-CoV-2 Pandemic
Source: Children (Basel). 2021 Aug 30;8(9):755. doi: 10.3390/children8090755 (PMC8471119; doi:10.3390/children8090755)
Supplement: Supplementary file 1 [file children-08-00755-s001.zip › children-1351129-supplementary.pdf]

Supplementary material

Table S1. Ad hoc questionnaire developed to assess the COVID-19 related parental stress

COVID-19 related parental stress
Total score (range 2-21)
Item 1 *How much is the COVID-19 pandemic a source of worry, anxiety or stress for you?* (1=not at all; 5= extremely)
Item 2 *How much has the COVID-19 pandemic negatively impacted your experience of becoming a parent?* (1=not at all; 5= extremely)
Item 3 *What factor(s) related to the COVID-19 pandemic negatively influenced the experience of becoming a parent?* (Yes or No)<br>a. Concerns for my health and that of my loved ones<br>b. Additional apprehension for my baby's health<br>c. Concerns about the future<br>d. Less time to spend with my baby due to COVID-19 preventive measures<br>e. Less physical contact with the baby due to COVID-19 preventive measures<br>f. Less support from relatives and friends due to COVID-19 preventive measures<br>g. Greater loneliness<br>h. Economic troubles<br>i. Work problems<br>l. Loss or illness<br>m. Other

Table S2. COVID-19 related parental stress

Variable	n (%) or median (IQR)
COVID-19 pandemic as source of stress:	
Not at all stressful	5 (11)
Mildly stressful	7 (16)
Moderately stressful	19 (44)
Very stressful	8 (18)
Extremely stressful	5 (11)
Negative impact of COVID-19 pandemic on becoming parent:	
No impact	9 (20)
Mild impact	7 (16)
Moderate impact	13 (30)
High impact	7 (16)
Very high impact	8 (18)
COVID-19 related factors having a negative impact on becoming parent:	
Less time spent with the infant	36 (82)
Less physical contact with the infant	32 (73)
Additional concerns about infant health	25 (57)
Less support from family and friends	18 (41)
Concerns about family/friends health	17 (39)
Loneliness	14 (32)
Concerns about the future	8 (18)
Economic troubles	6 (14)
Family loss or serious illness	4 (9)
Other stressful factors*	4 (9)
Work problems	3 (7)
Overall score on COVID-19 related parental stress, ranging from 2 (no stress) to 21 (very high stress)	10 (8-12)

* Parents reported as “other stressful factors” the inability to share visits to the child in the NICU with the partner.

Table S3. Factors associated with overall score on COVID-19 related parental stress

Variable	Overall score on COVID-19 related parental stress: median (IQR) or Spearman correlation coefficient	p-value
Parents:<br>Mothers<br>Fathers	 10 (8-13)<br>9 (7-11)	0.24
Parental age, years	-0.11	0.47
Education level:<br>Primary or middle school<br>High school<br>University	 12 (9-15)<br>9 (7-10)<br>10 (8-11)	0.09
Number of pregnancies:<br>One pregnancy<br>Two pregnancies	 10 (8-12)<br>10 (8-12)	0.91
Visits to the infant in the NICU, n	-0.15	0.32
STAI state	0.44	0.003
STAI trait	0.46	0.002
EPDS	0.35	0.02
PSS-SS	0.34	0.02
PSS-IBA	0.28	0.07
PSS-PRA	0.38	0.01
PSS-perceived stress item	0.43	0.003
PSS-total score	0.38	0.01
Gestational age, weeks	-0.07	0.64
Birth weight, grams	-0.06	0.70
Age at admission, days	-0.11	0.45
Delivery:<br>Vaginal or elective caesarean<br>Emergency caesarean	 11 (9-13)<br>9 (7-11)	0.17

Table S4. Association between the main COVID-19 related factors having a negative impact on becoming parent and STAI trait and EPDS

COVID-19 related factors having a negative impact on becoming parent	STAI trait		EPDS	
	Median (IQR)	p-value	Median (IQR)	p-value
Less time spent with the infant:<br>Disagree<br>Agree	 40 (30-50)<br>40 (35-45)	0.81	 11 (8-13)<br>9 (6-12)	0.32
Less physical contact with the infant:<br>Disagree<br>Agree	 30 (27-41)<br>41 (36-46)	0.03	 8 (6-12)<br>10 (7-12)	0.30
Concerns about infant health:<br>Disagree<br>Agree	 36 (31-40)<br>42 (36-47)	0.04	 9 (6-12)<br>9 (7-13)	0.35

Table S5. Comparison of mother-father pairs

	Mothers (n=19)	Fathers (n=19)	p-value
Visits to the infant in the NICU, n	7 (5-12)	6 (4-13)	0.81
Overall score on Covid-19 related parental stress [a]	10 (8-13)	9 (7-11)	0.10
STAI state	44 (36-50)	40 (36-50)	0.78
STAI trait	40 (35-45)	37 (34-43)	0.67
EPDS	10 (7-13)	7 (6-9)	0.02
PSS-SS	1.8 (1.6-2.6)	1.7 (1.3-1.8)	0.34
PSS-IBA	1.9 (1.4-2.4)	1.6 (1.3-1.9)	0.01
PSS-PRA	3.3 (2.7-3.8)	2.6 (2.1-3.0)	0.02
PSS-perceived stress item	4 (3-4)	3 (3-4)	0.75
PSS total score	2.4 (2.1-2.8)	2.0 (1.6-2.2)	0.01

Data expressed as median (IQR).
